# Biomechanical evaluation of the spring ligament and the posterior tibial tendon by shear-waves elastography: validation of a reliable and reproducible measurement protocol

**DOI:** 10.1186/s40634-023-00678-w

**Published:** 2023-11-25

**Authors:** Grégoire Rougereau, Thibault Marty-Diloy, Marie Vigan, Kalinka Donadieu, Raphaël Vialle, Tristan Langlais, Alexandre Hardy

**Affiliations:** 1 Department of Pediatric Orthopedic Surgery, Sorbonne University, Armand Trousseau Hospital, APHP, 75571 Paris, France; 2 Department of Adult Orthopedic Surgery, Sorbonne University, Pitié-Salpêtrière Hospital, APHP, 75571 Paris, France; 3 Unité de Recherche Clinique Hôpitaux Universitaires Paris Ile-de-France Ouest, APHP, 92100 Boulogne-Billancourt, France; 4 Department for Innovative Therapies, Musculoskeletal Disease Sorbonne University, The MAMUTH Hospital University, Paris, France; 5 Department of Pediatric Orthopedic Surgery, Toulouse University, Children’s Hospital, Purpan, Toulouse, France; 6 Department of Orthopedic Surgery, Clinique du Sport, 75005 Paris, France

**Keywords:** Elasticity imaging techniques, Elastography, Feasibility study, Posterior Tibial Tendon, Spring ligament

## Abstract

**Purpose:**

The anatomy of the spring ligament complex, as well as its pathology, is not well known in daily clinical practice. The purpose of this study was to evaluate the shear-wave elastography properties of the spring ligament and the posterior tibial tendon in healthy adults, and to assess the reliability and reproducibility of these measurements.

**Methods:**

Shear-wave elastography was used to evaluate both ankles in 20 healthy patients (10 females/10 males) resting on a hinge support with their ankles in neutral, valgus 20° and varus 30° positions. The stiffness of the spring ligament and posterior tibial tendon was assessed by measuring the speed of shear wave propagation through each structure.

**Results:**

Posterior tibial tendon and spring ligament reach a maximum estimated stiffness in valgus 20° position (7.43 m/s vs 5.73 m/s, respectively). Flat feet were associated with greater spring ligament stiffness in the 20° valgus position (*p* = 0.01), but not for the posterior tibial tendon (*p* = 0.71). The physiologic weightbearing hindfoot attitude had no impact on the stiffness of the posterior tibial tendon or the spring ligament, regardless of the analysis position. Intra- and inter-observer agreements were all excellent for spring ligament stiffness, regardless of ankle position, and were good or excellent for posterior tibial tendon.

**Conclusions:**

This study describes a protocol to assess the stiffness of tibialis posterior and the spring ligament by shear-wave elastography, which is reliable, reproducible, and defines a corridor of normality. Further studies should be conducted to define the role of elastography for diagnosis/ evaluation of pathology, follow-up, or surgical strategies.

## Introduction

The spring ligament inserts between the sustentaculum tali and the navicular, and is composed of 3 bundles: the superomedial calcaneonavicular ligament, the longitudinal inferoplantar calcaneonavicular ligament, and the oblique medioplantar calcaneonavicular ligament (or third ligament) [4, 7, 19, 20]. This structure plays a static support role keeping the internal arch of the foot open, working in synergy with the posterior tibial tendon (PTT) which plays a dynamic support role [14]. Although isolated spring ligament injuries exist, they are most often associated with those of the PTT [3] leading to the development of a flat foot.

The reference examination for studying the spring ligament is MRI [27]. An alternative to MRI is the use of ultrasound, which allows precise visualization of the superomedial bundle with a better spatial resolution than MRI, especially in its relationship with the PTT [11, 12]. Nevertheless, it does not allow analysis of the longitudinal infero-plantar and oblique medio-plantar bundles, and may be limited by its operator-dependent nature [11, 12], although this point is debated [28].

Shear-waves elastography (SWE) is a non-invasive ultrasound technique that studies quantitatively the mechanical properties of tissues by shear wave speed propagation [9]. It is used to measure the shear-wave speed (SWS) within the soft tissues, its speed being all the greater as the rigidity of the structures is high. It is regularly used in orthopaedics to study muscles [17, 21], tendon structures [11], or even ankle ligaments [23, 24].

To date, there are no tools for quantitatively assessing the biomechanical properties of these independent structures in vivo. No elastographic data currently exists to describe these ligamentous and tendinous structures. Thus, normal SWE values for these structures, is representative of the different morphotypes of the hindfoot and arch of the foot, do not exist. Nevertheless, the SWE signal may be unstable depending on the anatomical structures analyzed and its interpretation may be influenced by the operator's experience [5]. A reproducible static and dynamic measurement protocol needs to be proposed, validated initially on asymptomatic healthy subjects.

The purpose of this study was to evaluate the spring ligament and PTT SWE properties in a cohort of healthy adult volunteers, and to assess the reliability and reproducibility of these measurements. The hypothesis was that SWE is a reliable means of examining the spring ligament, and that it was associated with a decrease in its stiffness in case of flatfoot morphology.

## Materials and methods

This is a prospective study that was conducted at a single centre, and analysed 20 consecutive healthy subjects. The study was approved by Ethical Committee (C.P.P Île de France IV: records 14,409).

### Subjects

Subjects were all resident medical officers, or medical students, of the department, and were enrolled in the study between November 2020 and January 2021. A questionnaire was performed to ensure the absence of any past medical history among: fracture, sprain, or ankle surgery. Before participation, all the volunteers were informed of the purpose of the study and consent were obtained. For each subject, both ankles were included: forty ankles in total were analysed. In addition, the following demographic data were recorded: age, sex, height, side of the dominant foot, type of foot arch (neutral/ flat/ high), and type of hind foot alignment (neutral/ varus/ valgus). These last two parameters were obtained using a podoscope: which uses the Chippaux-Smirak index (CSI) to classify the type of plantar arch and the standing tibiocalcaneal angle to classify the type of hind foot alignment [2, 6].

### Population

Twenty consecutive subjects (10 females, 10 males), with mean aged 25.2 (range from 22 to 31 years old), were enrolled. The average weight was 61.9 ± 14.6 kg, and height 1.72 ± 0.09 m. Each subject was examined bilaterally, producing a total of 40 ankles for assessment. Twenty-foot arches were normal, 12 flat feet and 8 high arches. The hindfoot axis was neutral in 11 cases, valgus in 21 cases and varus in 8 cases. All subjects were asymptomatic at the time of examination. Those with valgus flatfoot were all flexible, presenting a type 1 CSI, with a moderate hindfoot valgus ranging from 7° to 12°.

### Shear-wave speed (SWS) acquisition protocol

An Aixplorer ultrasound scanner (Supersonic Imagine, Aix-en-Provence, France), with a 20.2 mm 8 MHz linear probe was used. The probe was applied to the medial ankle surface anterior and inferior to the medial malleolus in front of the head of the talus. The probe was first positioned in the axis of PTT, then moved downward until the PTT was sliding on the superomedial bundle on the same image. The superomedial bundle was the only part of the spring ligament analyzed in this study because it is the main biomechanical active bundle [1], and because it is the only part that can be reliably and reproducibly identified by ultrasound [11]. For all acquisitions, the probe was applied perpendicularly to the skin and the pressure applied was as light as possible during the acquisition, in order to limit elasticity variation and tissue deformation [15].

**Fig. 1:**
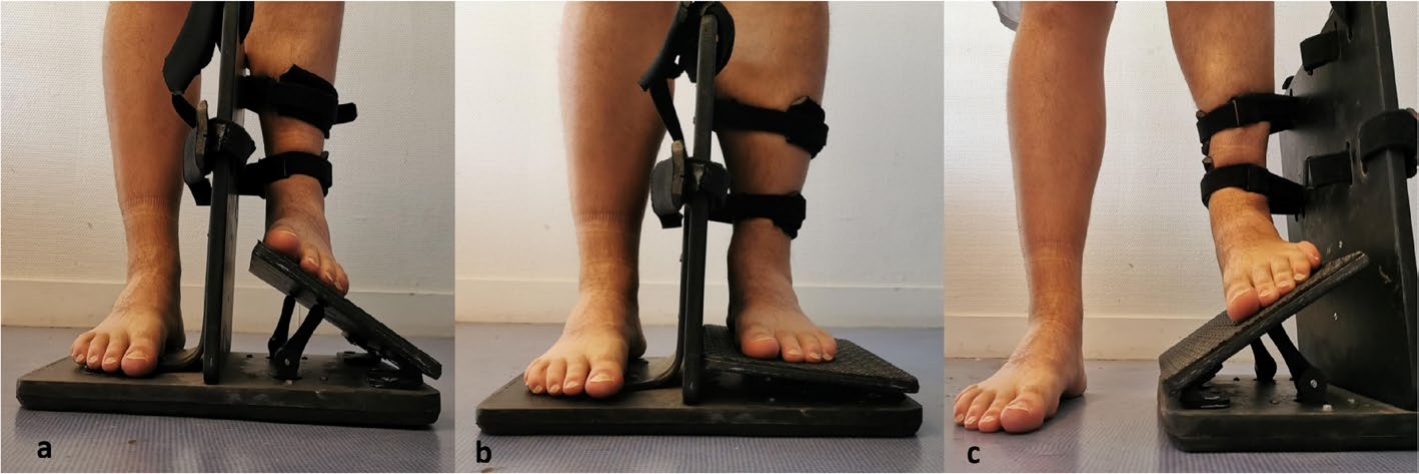
Ankle examination protocol. The patient was in full support on the hinge plate in 30° varus position (**a**), neutral position (**b**), and 20° valgus position (**c**)

Patients were positioned standing on an articulated platelet (Proteor®, Paris, France). The examination was performed with full weight bearing with ankle in neutral position, valgus 20° and varus 30° (Fig. 1). Two orthopedic surgeons, previously trained in ultrasound, realised blind data acquisition and repeated each hindfoot standing position three times, as per the protocol, for each patient, side and position three times as per the protocol.

### Measurements

Shear modulus (µ = ρ·SWS^2^) was calculated by the machine, where ρ is the soft tissue mass density. Each acquisition was in a 15 s video clip, from which the most stable signal image was frozen. Then, the operator defined a round region of interest (ROI) with a fixed two millimeters diameter for PTT and three millimeters diameter for spring ligament positioned in the middle of each ligament. The mean shear modulus ($\overline{\upmu }$) was then calculated automatically. Areas near bony structures were excluded from the measurement because of the risk of artefacts. This measurement protocol was performed for each acquisition, and repeated three times by each examiner to obtain an average.

### Reliability and statistics

The Wilcoxon-Mann Whitney for unpaired data were used to analyze the association between the different variables. The Krsukall-Wallis H-test for independent groups was used to compare the samples' median values, and a Dunn’s multiple comparison test was then performed. The alpha risk was set to 0.05. The intra-class correlation coefficient (ICC) was used to evaluate the intra-rater and inter-rater agreements in SWS stiffness measurements. The Bland–Altman method was used to visualize means in measurements between raters versus corresponding differences in measurements between raters and to compute the 95% limits of agreements between raters. An ICC value below 0.4 was considered as a poor agreement, between 0.4 and 0.75 as good agreement, and above 0.75 as excellent agreement [16]. A SWS normally corridor was calculated as the range [5^th^-95^th^ percentile] for each ligament. The determination of the sample size indicates that 38 samples are required to have a 90% chance of correctly rejecting the null hypothesis of *r* = 0 at the 0.05 significance level assuming a linear correlation of 0.5 (moderate correlation) between spring ligament and posterior tibial tendon. Statistical analysis was performed using the R Language and Environment for Statistical Computing version 3.6.3 (R Core Team, version 2020, Vienna, Austria).

## Results

### Inter and intra observers’ reproducibility

Intra- and inter-observer agreements were all excellent for spring ligament stiffness, regardless of ankle position. Intra- and inter-observer agreements were good or excellent for PTT stiffness evaluation depending positions. All inter- and intra-class correlations are summarized in Table 1.

**Table 1:** Inter and intra-agreement correlation using isolated measurement comparison or the average of three measurements comparison

	**Isolated measurement comparison**		**Averages of 3 measurements comparison**		
	**ICC first observer**	**ICC second observer**	**ICC first observer**	**ICC second observer**	**ICC inter observer**
**Spring Ligament**					
**Normal**	0.77 [0.68–0.85]	0.82 [0.75–0.89]	**0.85 [0.76–0.91]**	**0.87 [0.82–0.93]**	0.88 [0.78–0.93]
**Valgus 20°**	0.82 [0.74–0.89]	0.79 [0.71–0.86]	**0.89 [0.82–0.94]**	**0.84 [0.75–0.91]**	0.87 [0.77–0.93]
**Varus 30°**	0.87 [0.82–0.92]	0.88 [0.82–0.92]	**0.92 [0.87–0.95]**	**0.94 [0.90–0.96]**	0.94 [0.88–0.97]
**Posterior tibial tendon**					
**Normal**	0.66 [0.55–0.77]	0.69 [0.58–0.79]	**0.76 [0.64–0.86]**	**0.85 [0.76–0.91]**	0.78 [0.62–0.88]
**Valgus 20°**	0.64 [0.53–0.75]	0.59 [0.47–0.71]	**0.77 [0.65–0.86]**	**0.67 [0.52–0.80]**	0.63 [0.41–0.79]
**Varus 30°**	0.62 [0.51–0.74]	0.59 [0.47–0.72]	**0.74 [0.61–0.84]**	**0.70 [0.55–0.81]**	0.86 [0.76–0.93]

### Biomechanical characteristics of the spring ligament complex during ankle mobilizations

**Fig. 2:**
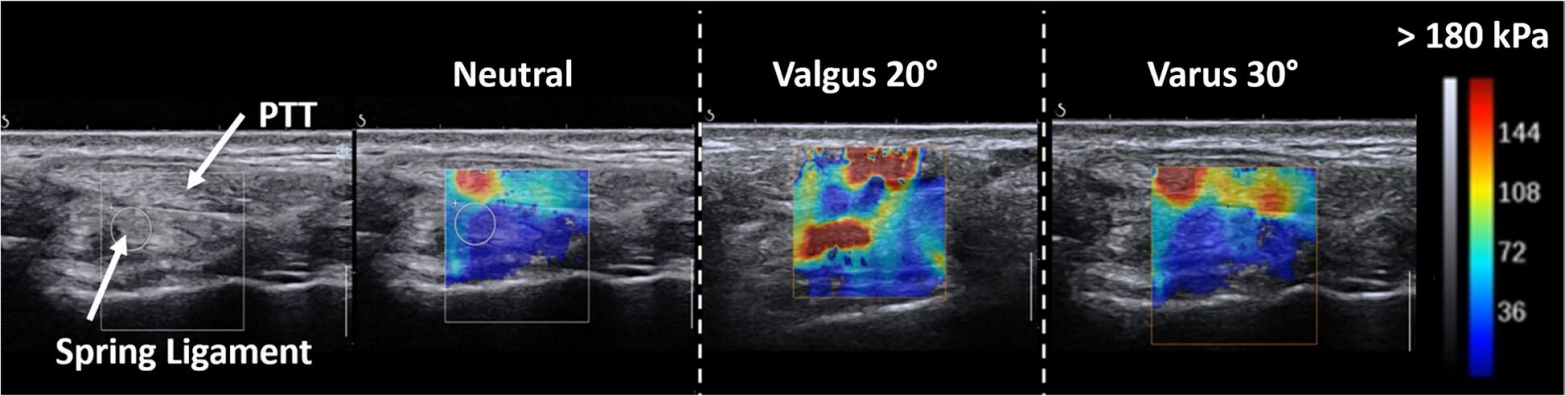
SWE examination for the Spring ligament and posterior tibialis tendon (PTT). Shear wave speed (SWS) was measured for each structure, with the stiffness scale provided on the right

**Fig. 3:**
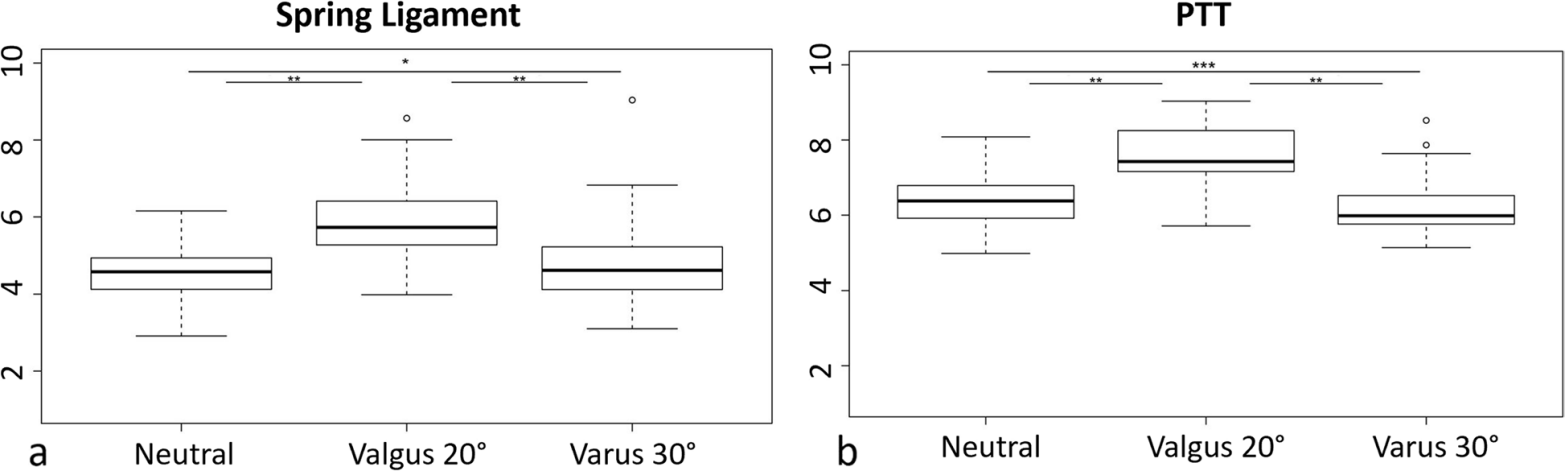
Normally corridor of stiffness assessed by SWE (m/s) with their 95% confidence interval for the Spring ligament (a), and PTT (b). *:*p* = 0.50; **:*p* < 0.0001; ***:*p* = 0.37

The median stiffness of PTT and spring ligament varies according to ankle position: PTT and spring ligament reach their maximum stiffness in valgus 20° position (7.43 m/s (range from 5.71 to 9.03); 5.73 m/s (range from 3.98 to 8.57), respectively). There were no differences in SWS measured in neutral and varus 30° position for PTT (*p* = 0.37) and for the spring ligament (*p* = 0.50). The variation of stiffness evaluated by SWS according to the ankle position is shown in Fig. 2. Finally, SWS values of PTT and spring ligament according to the ankle position are reported in Fig. 3.

### Impact of demographic characteristics

No demographic data (age, sex, dominant side, height) had an impact on spring ligament stiffness as assessed by SWE. PTT SWS decreased with age (*ρ* = -0.34; *p* = 0.03) and with male gender (*p* = 0.03). The impact of demographics on spring ligament complex stiffness is reported in Table 2.

**Table 2:** Impact of demographic data (sex, age, height, dominant side) on the stiffness of each structure in neutral position and then in functional position

	**Gender**			**Dominant side**			**Correlation (ρ) with**			
	**Male**	**Female**	***p value***	**Yes**	**No**	***p value***	**Age**	***p value***	**Height**	***p value***
**Spring ligament**										
**Normal**	4.58 ± 1.01	4.46 ± 0.62	0.56	4.54 ± 0.88	4.58 ± 0.81	0.94	-0.26	0.11	0.04	0.83
**Valgus 20°**	6.09 ± 1.2	5.65 ± 0.65	0.21	5.9 ± 1.07	6.1 ± 1.15	0.78	-0.24	0.14	0.24	0.14
**Varus 30°**	4.7 ± 1.03	4.79 ± 1.28	0.69	4.91 ± 1.34	4.61 ± 0.91	0.52	-0.31	**0.05**	-0.17	0.30
**Posterior tibialis tendon (PTT)**										
**Normal**	6.3 ± 0.79	6.38 ± 0.72	0.66	6.47 ± 0.81	6.51 ± 0.73	0.61	-0.16	0.32	0.006	0.97
**Valgus 20°**	7.66 ± 1.01	7.5 ± 0.86	0.33	7.63 ± 0.93	7.61 ± 0.96	0.76	-0.30	0.06	0.08	0.62
**Varus 30°**	6.01 ± 0.61	6.43 ± 0.9	**0.03**	6.19 ± 0.86	6.39 ± 0.75	0.68	-0.34	**0.03**	-0.26	0.11

Flat-foot morphology was associated with a significant increase in spring ligament stiffness in the 20° valgus position (*p* = 0.01), but had no impact on PTT. Hindfoot type had no impact on variation in PTT and spring ligament stiffness. The impact of foot morphological data on ligament complex stiffness is presented in Table 3.

**Table 3:** Impact of foot and ankle morphology on the stiffness of each structure in neutral position and then in functional position

	**Foot arch**				**Hind foot**			
	**High arch**	**Neutral**	**Flat foot**	***p value***	**Varus**	**Neutral**	**Valgus**	***p value***
**Spring ligament**								
**Normal**	4.56 ± 0.57	4.29 ± 0.95	4.93 ± 0.63	0.19	4.42 ± 0.65	4.49 ± 0.75	4.61 ± 0.93	0.96
**Valgus 20°**	5.5 ± 0.69	5.68 ± 1.09	6.64 ± 1.09	**0.01**	5.64 ± 1.01	6.49 ± 1.1	5.74 ± 1.07	0.13
**Varus 30°**	4.34 ± 0.82	4.76 ± 1.32	5.05 ± 0.89	0.22	4.75 ± 0.76	5.28 ± 1.34	4.5 ± 0.93	0.19
**Posterior tibialis tendon (PTT)**								
**Normal**	6.61 ± 0.61	6.3 ± 0.86	6.42 ± 0.69	0.41	6.62 ± 1.02	6.47 ± 0.78	6.28 ± 0.66	0.58
**Valgus 20°**	7.47 ± 0.96	7.66 ± 0.95	7.57 ± 0.92	0.71	7.52 ± 0.88	7.91 ± 0.96	7.47 ± 0.92	0.47
**Varus 30°**	6.53 ± 0.92	6.23 ± 0.8	5.91 ± 0.65	0.14	6.51 ± 1.06	6.24 ± 0.92	6.08 ± 0.61	0.57

Finally, there was a statistical correlation in the stiffness of the spring ligament and the PTT in varus 30° (*ρ* = 0.43; *p* = 0.006). There was no correlation in stiffness between the two structures in other positions (Table 4).

**Table 4:** Inter structure correlation for each analyzed position

	**Rho**	***p value***
**Correlation between Spring ligament and PTT**		
Normal	-0.09	0.58
Valgus 20°	0.23	0.16
Varus 30°	0.43	**0.006**

## Discussion

The main results of this study are that the SWE is reliable and reproducible to analyze the stiffness of the superomedial calcaneonavicular ligament spring ligament and posterior tibial tendon. Moreover, it confirms the mechanical action of the spring ligament in the ankle valgus position.

This study only focused on the main ligament of the spring ligament: the superomedial calcaneonavicular ligament. It is the widest and thickest of the three ligaments [1, 20]. Locally, it stabilizes the medial arch of the ankle with the PTT passing over its superficial medial surface, and the anterior part of the superficial bundle of the medial collateral ligament with which it shares fibers [1, 10]. The superomedial ligament is the strongest of the spring ligament bundles: with a breaking load of 665.5N compared to 291.3N for the lower bundle [1]. Reeck et al. have shown that it is important to analyze the spring ligament complex in its dynamic component [22]. During walking, the stresses under the talus are mainly distributed by the subtalar and talonavicular joints: only 9% are absorbed by the spring ligament complex [22]. Although this contribution is small, it is still critical because it is the only internal structure that allows the medial stresses of the talus head to be accommodated. Isolated section of the PTT was not sufficient to induce a flat foot, whereas section of the spring ligament alone immediately induced an intermediate flat foot [14]. This point was confirmed by Masaragian et al. who report a series of ten patients with acquired flatfoot deformity due to isolated spring ligament injury without tibialis posterior tendon tear [18]. Furthermore, no variation in PTT muscle stiffness was found between flat-footed deformities and healthy subjects [17]. These two structures therefore have their own actions, which is confirmed by our study, which found no correlation between the evolution of their rigidities in the functional position.

Various radiological or anatomical studies have examined the impact of certain demographic criteria on the morphology of the spring ligament. No correlation was found between the thickness of the superomedial ligament and the PTT, nor with age [19] or BMI [27]. By MRI, the superomedial ligament appears thinner in women than in men [19]. The results of this study complement these initial data by showing that none of these factors play a role in the stiffness of the elastic ligament, regardless of hindfoot position.

The reference imaging to assess the spring ligament complex is MRI [19], because it allows a slightly better view of the other bundles than the superomedial. Even if it has a better definition than ultrasound, it is not perfect: MRI identifies the longitudinal inferoplantar ligament in 91% of cases, and the oblique medioplantar in only 77% of cases [19]. Moreover, its discriminating power in case of spring ligament injury is disappointing, with a sensitivity of 54–77% and a specificity of 100% [26]. Ultrasound has the advantage of being easy to access, and of allowing a dynamic examination and a better spatial resolution [11–13]. Its diagnosis is made in the presence of a heterogeneous signal, hyperthrophy or atrophy, or the presence of a visible rupture [11]. Its sensitivity is 87.5% but using MRI as a reference, which has already shown some limitations [26]. Their limitation for the study of the spring ligament is their operator-dependent nature, and the absence of quantitative values for monitoring ligament status. Our study reports that SWE is reliable and reproducible, defining a corridor of normality in dynamic maneuvers. In order to take this study further, a cohort of patients with spring ligament injuries could be studied, to help understand the use of SWE in clinical practice for the diagnosis and even the prognosis of these injuries with the help of a quantitative evaluation of its stiffness.

The surgical management remains debated and several techniques of spring ligament reconstruction have been described as alternatives to triple arthrodesis. There is great variability in repair or reconstruction techniques [3, 8, 25]. Whatever the technique used, reproduction of the physiological stiffness of the native elastic ligament may be the key. The SWE could be of use to evaluate the stiffness and biomechanical properties of the different reconstructions intraoperatively, then over time, to approximate normal values. This in vivo and reproducible measurement tool could be a dynamic monitoring tool during postoperative surveillance, complementary to MRI, to control the evolution of the stiffness of the reconstruction, in particular the risk of loosening over time.

This study has limitations. First of all, it is a preliminary study on a limited number of healthy subjects, which limits its short-term significance. Additionally, this study analysed only the most superficial bundle of the spring ligament: the superomedial calcaneonavicular ligament. This was chosen as the superomedial bundle is the biomechanically active part of the spring ligament. It has been shown that the analysis of other bundles is more difficult, and not very accessible in current practice [11]. Despite the validation of this protocol, there may still be variability in the pressure applied by the operator to the probe, which could be a limitation for non-professional examiners [15]. Finally, the SWE signal of the PTT was more unstable than that of the spring ligament, which may discuss the use of this tool for this structure.

## Conclusion

The spring ligament, the main stabilizer of the medial arch of the foot, has an important dynamic function. This study describes a protocol to assess its stiffness by SWE, which is reliable, reproducible, and defines a corridor of normality. Flat-foot morphology was associated with a significant increase in spring ligament stiffness in valgus position, but had no impact on PTT. Further studies could be conducted to define its role in diagnosis, follow-up or surgical reconstruction strategies.

## Data Availability

The datasets used and/or analysed during the current study are available from the corresponding author on reasonable request.

## References

[1] Davis WH, Sobel M, DiCarlo EF, Torzilli PA, Deng X, Geppert MJ, Patel MB, Deland J (1996). Gross, histological, and microvascular anatomy and biomechanical testing of the spring ligament complex. Foot Ankle Int.

[2] de Cesar NC, Kunas C, Soukup D, Marinescu A, Ellis S (2018). Correlation of clinical evaluation and radiographic hindfoot alignment in stage II adult-acquired flatfoot deformity. Foot Ankle Int.

[3] Deland JT, Arnoczky SP, Thompson FM (1992). Adult acquired flatfoot deformity at the talonavicular joint: reconstruction of the spring ligament in an in vitro model. Foot Ankle.

[4] Döring S, Provyn S, Marcelis S, Shahabpour M, Boulet C, de Mey J, De Smet A, De Maeseneer M (2018). Ankle and midfoot ligaments: Ultrasound with anatomical correlation: a review. Eur J Radiol.

[5] Dubois G, Kheireddine W, Vergari C, Bonneau D, Thoreux P, Rouch P, Tanter M, Gennisson J-L, Skalli W (2015). Reliable protocol for shear wave elastography of lower limb muscles at rest and during passive stretching. Ultrasound Med Biol.

[6] Fascione JM, Crews RT, Wrobel JS (2012). Dynamic footprint measurement collection technique and intrarater reliability: ink mat, paper pedography, and electronic pedography. J Am Podiatr Med Assoc.

[7] Flores DV, Mejía Gómez C, Fernández Hernando M, Davis MA, Pathria MN (2019). Adult acquired flatfoot deformity: anatomy, biomechanics, staging, and imaging findings. Radiographics.

[8] Fogleman JA, Kreulen CD, Sarcon AK, Michelier PV, Giza E, Doty JF (2021). Augmented spring ligament repair in pes planovalgus reconstruction. J Foot Ankle Surg.

[9] Gennisson JL, Deffieux T, Fink M, Tanter M (2013). Ultrasound elastography: Principles and techniques. Diagn Interv Imaging.

[10] Golanó P, Vega J, de Leeuw PA, Malagelada F, Manzanares MC, Götzens V, van Dijk CN (2016). Anatomy of the ankle ligaments: a pictorial essay. Knee Surg Sports Traumatol Arthrosc.

[11] Hardy A, Rodaix C, Vergari C, Vialle R (2017). Normal range of patellar tendon elasticity using the sharewave elastography technique: An in vivo study in normal volunteers. Surg Technol Int.

[12] Harish S, Jan E, Finlay K, Petrisor B, Popowich T, Friedman L, Wainman B, Jurriaans E (2007). Sonography of the superomedial part of the spring ligament complex of the foot: a study of cadavers and asymptomatic volunteers. Skeletal Radiol.

[13] Harish S, Kumbhare D, O’Neill J, Popowich T (2008). Comparison of Sonography and Magnetic Resonance Imaging for Spring Ligament Abnormalities. J Ultrasound Med.

[14] Jennings MM, Christensen JC (2008). The effects of sectioning the spring ligament on Rearfoot stability and posterior Tibial tendon efficiency. J Foot Ankle Surg.

[15] Kot BCW, Zhang ZJ, Lee AWC, Leung VYF, Fu SN (2012). Elastic modulus of muscle and tendon with shear wave ultrasound elastography: variations with different technical settings. PLoS One.

[16] Landis JR, Koch GG (1997). The measurement of observer agreement for categorical data. Biometrics.

[17] Marouvo J, Sousa F, André MA, Castro MA (2023). Tibialis posterior muscle stiffness assessment in flat foot subjects by ultrasound based Shear-Wave Elastography. Foot (Edinb).

[18] Masaragian HJ, Massetti S, Perin F, Coria H, Cicarella S, Mizdraji L, Rega L (2020). flatfoot deformity due to isolated spring ligament injury. J Foot Ankle Surg.

[19] Mengiardi B, Zanetti M, Schöttle PB, Vienne P, Bode B, Hodler J, Pfirrmann CW (2005). Spring ligament complex: MR imaging-anatomic correlation and findings in asymptomatic subjects. Radiology.

[20] Patil V, Ebraheim NA, Frogameni A, Liu J (2007). Morphometric Dimensions of the Calcaneonavicular (Spring) Ligament. Foot Ankle Int.

[21] Pietton R, David M, Hisaund A, Langlais T, Skalli W, Vialle R, Vergari C (2021). Biomechanical evaluation of intercostal muscles in healthy children and adolescent idiopathic scoliosis: a preliminary study. Ultrasound Med Biol.

[22] Reeck J, Felten N, McCormack AP, Kiser P, Tencer AF, Sangeorzan BJ (1998). Support of the talus: a biomechanical investigation of the contributions of the talonavicular and talocalcaneal joints, and the superomedial calcaneonavicular ligament. Foot Ankle Int.

[23] Rougereau G, Langlais T, Vigan M, Hardy A, Vialle R, Marty-Diloy T, Cambon-Binder A (2022). Ankle syndesmosis biomechanical evaluation by shear-waves elastography in healthy young adults: Assessment of the reliability and accuracy of the measurements and definition of a corridor of normality. Foot Ankle Surg.

[24] Rougereau G, Marty-Diloy T, Vigan M, Donadieu K, Hardy A, Vialle R, Langlais T (2022). A Preliminary Study to Assess the Relevance of Shear-Wave Elastography in Characterizing Biomechanical Changes in the Deltoid Ligament Complex in Relation to Ankle Position. Foot Ankle Int.

[25] Tan GJ, Kadakia AR, Ruberte Thiele RA, Hughes RE (2010). Novel reconstruction of a static medial ligamentous complex in a flatfoot model. Foot Ankle Int.

[26] Toye LR, Helms CA, Hoffman BD, Easley M, Nunley JA (2005). MRI of spring ligament tears. AJR Am J Roentgenol.

[27] Yao L, Gentili A, Cracchiolo A (1999). MR imaging findings in spring ligament insufficiency. Skeletal Radiol.

[28] Youk JH, Son EJ, Park AY, Kim JA (2014). Shear-wave elastography for breast masses: local shear wave speed (m/sec) versus Young modulus (kPa). Ultrasonography.

